# 1,5-Bis[(*E*)-cyclo­pentyl­idene]thio­carbono­hydrazide

**DOI:** 10.1107/S1600536809009325

**Published:** 2009-03-25

**Authors:** Qingliang Guo, Junshan Sun, Jikun Li, Rentao Wu, Wenzeng Duan

**Affiliations:** a Department of Chemistry and Environmental Science, Taishan University, 271021 Taian, Shandong, People’s Republic of China; b Department of Materials and Chemical Engineering, Taishan University, 271021 Taian, Shandong, People’s Republic of China

## Abstract

In the title mol­ecule, C_11_H_18_N_4_S, an intra­molecular N—H⋯N hydrogen bond [N⋯N = 2.558 (3)Å] is observed. The two cyclo­pentyl rings are disordered between two conformations in 1:1 and 2:1 ratios. In the crystal structure, weak inter­molecular N—H⋯S hydrogen bonds [N⋯S = 3.547 (3) Å] link pairs of mol­ecules into centrosymmetric dimers.

## Related literature

For related Schiff base derivatives of thio­carbohydrazide, see: Bacchi *et al.* (1996 ▶); Chantrapromma *et al.* (2001 ▶).
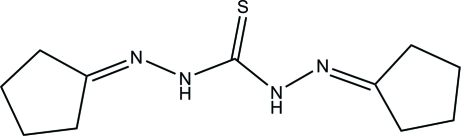


## Experimental

### 

#### Crystal data

C_11_H_18_N_4_S
*M* *_r_* = 238.35
Triclinic, 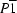

*a* = 6.0344 (19) Å
*b* = 10.114 (3) Å
*c* = 11.137 (3) Å
α = 106.579 (5)°
β = 96.897 (5)°
γ = 100.574 (5)°
*V* = 629.6 (3) Å^3^
*Z* = 2
Mo *K*α radiation
μ = 0.24 mm^−1^
*T* = 273 K
0.12 × 0.08 × 0.06 mm

#### Data collection

Bruker SMART APEX diffractometer
Absorption correction: multi-scan (*SADABS*; Sheldrick, 1996 ▶) *T*_min_ = 0.972, *T*_max_ = 0.986
3340 measured reflections
2212 independent reflections
1673 reflections with *I* > 2σ(*I*)
*R*_int_ = 0.013

#### Refinement

*R*[*F* ^2^ > 2σ(*F* ^2^)] = 0.042
*wR*(*F* ^2^) = 0.112
*S* = 1.03
2212 reflections
172 parameters
53 restraints
H-atom parameters constrained
Δρ_max_ = 0.16 e Å^−3^
Δρ_min_ = −0.17 e Å^−3^

### 

Data collection: *SMART* (Siemens, 1996 ▶) ; cell refinement: *SAINT* (Siemens, 1996 ▶); data reduction: *SAINT* (Siemens, 1996 ▶); program(s) used to solve structure: *SHELXS97* (Sheldrick, 2008 ▶); program(s) used to refine structure: *SHELXL97* (Sheldrick, 2008 ▶); molecular graphics: *SHELXTL* (Sheldrick, 2008 ▶); software used to prepare material for publication: *SHELXTL* (Sheldrick, 2008 ▶).

## Supplementary Material

Crystal structure: contains datablocks I, global. DOI: 10.1107/S1600536809009325/cv2503sup1.cif

Structure factors: contains datablocks I. DOI: 10.1107/S1600536809009325/cv2503Isup2.hkl

Additional supplementary materials:  crystallographic information; 3D view; checkCIF report

## Figures and Tables

**Table 1:** Hydrogen-bond geometry (Å, °)

*D*—H⋯*A*	*D*—H	H⋯*A*	*D*⋯*A*	*D*—H⋯*A*
N3—H3⋯N2	0.86	2.17	2.558 (3)	108
N1—H1⋯S1^i^	0.86	2.70	3.547 (3)	170

Symmetry code: (i) 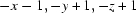.

**Fig. 1.**
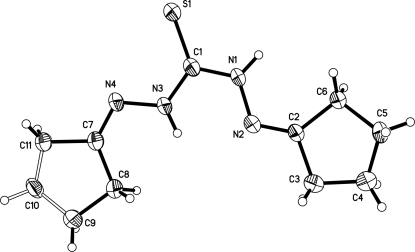
The molecular structure of the title compound showing the atomic numbering and 30% probability displacement ellipsoids. Only major parts of disordered rings are shown.

## supplementary crystallographic information

### Comment

Thiocarbohydrazide and its Schiff base derivatives have attracted considerable interest in the chemistry of metal complexes containing nitrogen and donors (Bacchi *et al.*, 1996; Chantrapromma *et al.*, 2001]. The interest in this field may be attributed to the striking structural features in the resultant metal complexes and their biological activities. Herein we present the synthesis and crystal structure of the title compound.

The title compound is shown in Fig. 1. Two cyclopentanone rings are disordered between two conformations in the ratios 1:1 and 2:1, respectively. The four N atoms and the C═S are almost coplanar with the mean deviation of 0.024 (2) Å. In this molecule, there exist intramolecular  N—H···N hydrogen bond (Table 1). Weak intermolecular N—H···S hydrogen bonds (Table 1) link two molecules into centrosymmetric dimers.

### Experimental

A solution of cyclopentanone and thiocarbohydrazide in ethanol in the ratio of 2:1 were refluxed for 8 h with stirring and cooled to the room temperature. The yellow precipitated powder of title compound was filtered and washed with water and ethanol, and then air dried thoroughly. A crystal suitable for X-ray diffraction was obtained by evaporation from a DMF and ethanol mixture. The yield is 78% and elemental analysis: calc. for C_11_H_18_N_4_S: C 55.43, H 7.61, N 23.51; found: C 55.26, H 7.49, N 23.88%. The elemental analyses were performed with PERKIN ELMER MODEL 2400 SERIES II. The CCDC number: 695533.

### Refinement

The H atoms were found in a difference map, then placed in idealized positions (C—H 0.97 Å, N—H 0.86 Å), and refined using a riding model, with *U*_iso_(H) = 1.2*U*_eq_(C,N). Two cyclopentanone rings were treated as disordered between two conformations with the refined occupancies 0.533 (14):0.567 (14) and 0.661 (14):0.339 (14), respectively.

### Crystal data

**Table:**

C_11_H_18_N_4_S	*Z* = 2
*M**_r_* = 238.35	*F*(000) = 256
Triclinic, *P*1	*D*_x_ = 1.257 Mg m^−^^3^
*a* = 6.0344 (19) Å	Mo *K*α radiation, λ = 0.71073 Å
*b* = 10.114 (3) Å	Cell parameters from 1126 reflections
*c* = 11.137 (3) Å	θ = 3.3–24.9°
α = 106.579 (5)°	µ = 0.24 mm^−^^1^
β = 96.897 (5)°	*T* = 273 K
γ = 100.574 (5)°	Block, colourless
*V* = 629.6 (3) Å^3^	0.12 × 0.08 × 0.06 mm

### Data collection

**Table:**

Bruker SMART APEX diffractometer	2212 independent reflections
Radiation source: fine-focus sealed tube	1673 reflections with *I* > 2σ(*I*)
graphite	*R*_int_ = 0.013
φ and ω scans	θ_max_ = 25.1°, θ_min_ = 1.9°
Absorption correction: multi-scan (*SADABS*; Sheldrick, 1996)	*h* = −6→7
*T*_min_ = 0.972, *T*_max_ = 0.986	*k* = −12→9
3340 measured reflections	*l* = −13→12

### Refinement

**Table:**

Refinement on *F*^2^	Secondary atom site location: difference Fourier map
Least-squares matrix: full	Hydrogen site location: inferred from neighbouring sites
*R*[*F*^2^ > 2σ(*F*^2^)] = 0.042	H-atom parameters constrained
*wR*(*F*^2^) = 0.112	*w* = 1/[σ^2^(*F*_o_^2^) + (0.0454*P*)^2^ + 0.1999*P*] where *P* = (*F*_o_^2^ + 2*F*_c_^2^)/3
*S* = 1.03	(Δ/σ)_max_ < 0.001
2212 reflections	Δρ_max_ = 0.16 e Å^−^^3^
172 parameters	Δρ_min_ = −0.17 e Å^−^^3^
53 restraints	Extinction correction: *SHELXL97* (Sheldrick, 2008), Fc^*^=kFc[1+0.001xFc^2^λ^3^/sin(2θ)]^-1/4^
Primary atom site location: structure-invariant direct methods	Extinction coefficient: 0.009 (3)

### Special details

**Table:**

Geometry. All e.s.d.'s (except the e.s.d. in the dihedral angle between two l.s. planes) are estimated using the full covariance matrix. The cell e.s.d.'s are taken into account individually in the estimation of e.s.d.'s in distances, angles and torsion angles; correlations between e.s.d.'s in cell parameters are only used when they are defined by crystal symmetry. An approximate(isotropic) treatment of cell e.s.d.'s is used for estimating e.s.d.'s involving l.s. planes.
Refinement. Refinement of *F*^2^ against ALL reflections. The weighted *R*-factor *wR* and goodness of fit *S* are based on *F*^2^, conventional *R*-factors *R* are based on *F*, with *F* set to zero for negative *F*^2^. The threshold expression of *F*^2^ > σ(*F*^2^) is used only for calculating *R*-factors(gt) *etc*. and is not relevant to the choice of reflections for refinement. *R*-factors based on *F*^2^ are statistically about twice as large as those based on *F*, and *R*- factors based on ALL data will be even larger.

### Fractional atomic coordinates and isotropic or equivalent isotropic displacement parameters (Å^2^)

**Table:**

	*x*	*y*	*z*	*U*_iso_*/*U*_eq_	Occ. (<1)
S1	−0.52345 (11)	0.37097 (7)	0.62060 (6)	0.0680 (3)	
N1	−0.1552 (3)	0.56488 (18)	0.63675 (17)	0.0545 (5)	
H1	−0.2334	0.5908	0.5814	0.065*	
N2	0.0700 (3)	0.63623 (19)	0.68996 (17)	0.0580 (5)	
N3	−0.1013 (3)	0.42144 (18)	0.75435 (17)	0.0580 (5)	
H3	0.0391	0.4685	0.7755	0.070*	
N4	−0.1709 (3)	0.3132 (2)	0.8044 (2)	0.0690 (6)	
C1	−0.2498 (4)	0.4543 (2)	0.6730 (2)	0.0509 (5)	
C2	0.1520 (3)	0.7462 (2)	0.6622 (2)	0.0517 (5)	
C3	0.3930 (4)	0.8296 (3)	0.7198 (3)	0.0774 (8)	
H3A	0.4066	0.8764	0.8103	0.093*	
H3B	0.5000	0.7683	0.7077	0.093*	
C4	0.4395 (15)	0.9365 (9)	0.6511 (10)	0.074 (2)	0.661 (14)
H4A	0.5092	0.9002	0.5781	0.089*	0.661 (14)
H4B	0.5411	1.0238	0.7073	0.089*	0.661 (14)
C5	0.2064 (11)	0.9613 (7)	0.6089 (10)	0.073 (2)	0.661 (14)
H5A	0.2052	0.9934	0.5347	0.088*	0.661 (14)
H5B	0.1645	1.0312	0.6768	0.088*	0.661 (14)
C6	0.0426 (4)	0.8155 (2)	0.5770 (2)	0.0603 (6)	
H6C	−0.1083	0.8253	0.5943	0.072*	0.661 (14)
H6B	0.0283	0.7611	0.4881	0.072*	0.661 (14)
H6A	−0.0701	0.8622	0.6154	0.072*	0.339 (14)
H6D	−0.0317	0.7467	0.4952	0.072*	0.339 (14)
C4'	0.409 (4)	0.9642 (13)	0.6854 (17)	0.074 (2)	0.339 (14)
H4C	0.5632	0.9997	0.6742	0.089*	0.339 (14)
H4D	0.3675	1.0367	0.7512	0.089*	0.339 (14)
C5'	0.241 (2)	0.9226 (17)	0.5619 (16)	0.073 (2)	0.339 (14)
H5D	0.3090	0.8804	0.4900	0.088*	0.339 (14)
H5C	0.1901	1.0042	0.5496	0.088*	0.339 (14)
C7	−0.0131 (4)	0.2930 (2)	0.8792 (2)	0.0588 (6)	
C8	0.2329 (4)	0.3678 (2)	0.9211 (2)	0.0607 (6)	
H8C	0.2503	0.4631	0.9781	0.073*	0.467 (14)
H8B	0.3050	0.3725	0.8487	0.073*	0.467 (14)
H8A	0.2478	0.4691	0.9564	0.073*	0.533 (14)
H8D	0.3131	0.3502	0.8497	0.073*	0.533 (14)
C9'	0.335 (2)	0.2757 (11)	0.9903 (7)	0.075 (3)	0.467 (14)
H9C	0.4080	0.2125	0.9337	0.090*	0.467 (14)
H9D	0.4496	0.3352	1.0638	0.090*	0.467 (14)
C10'	0.1445 (17)	0.1908 (17)	1.0324 (14)	0.079 (3)	0.467 (14)
H10A	0.1068	0.2460	1.1107	0.095*	0.467 (14)
H10B	0.1815	0.1049	1.0429	0.095*	0.467 (14)
C11'	−0.0457 (17)	0.1591 (6)	0.9191 (7)	0.070 (3)	0.467 (14)
H11C	−0.1947	0.1388	0.9428	0.083*	0.467 (14)
H11D	−0.0333	0.0784	0.8501	0.083*	0.467 (14)
C9	0.3302 (17)	0.3089 (6)	1.0216 (7)	0.0582 (18)	0.533 (14)
H9A	0.4817	0.2934	1.0116	0.070*	0.533 (14)
H9B	0.3374	0.3713	1.1070	0.070*	0.533 (14)
C10	0.1582 (12)	0.1699 (13)	0.9944 (14)	0.079 (3)	0.533 (14)
H10C	0.1655	0.1382	1.0689	0.095*	0.533 (14)
H10D	0.1894	0.0975	0.9242	0.095*	0.533 (14)
C11	−0.0769 (13)	0.1980 (11)	0.9599 (10)	0.066 (2)	0.533 (14)
H11A	−0.1893	0.1116	0.9111	0.079*	0.533 (14)
H11B	−0.1325	0.2467	1.0346	0.079*	0.533 (14)

### Atomic displacement parameters (Å^2^)

**Table:**

	*U*^11^	*U*^22^	*U*^33^	*U*^12^	*U*^13^	*U*^23^
S1	0.0570 (4)	0.0775 (5)	0.0726 (4)	−0.0016 (3)	−0.0087 (3)	0.0472 (4)
N1	0.0521 (11)	0.0585 (11)	0.0601 (11)	0.0071 (9)	−0.0029 (9)	0.0385 (9)
N2	0.0501 (11)	0.0654 (12)	0.0667 (12)	0.0089 (9)	−0.0001 (9)	0.0407 (10)
N3	0.0523 (11)	0.0579 (11)	0.0695 (12)	0.0014 (9)	−0.0062 (9)	0.0425 (10)
N4	0.0609 (12)	0.0639 (12)	0.0878 (14)	−0.0038 (9)	−0.0116 (11)	0.0541 (11)
C1	0.0573 (13)	0.0515 (12)	0.0478 (12)	0.0109 (10)	0.0021 (10)	0.0257 (10)
C2	0.0464 (12)	0.0609 (13)	0.0560 (13)	0.0100 (10)	0.0036 (10)	0.0348 (11)
C3	0.0524 (15)	0.0933 (19)	0.0922 (19)	0.0008 (13)	−0.0100 (13)	0.0573 (16)
C4	0.056 (3)	0.070 (3)	0.095 (5)	−0.001 (2)	−0.002 (3)	0.040 (4)
C5	0.064 (3)	0.070 (3)	0.093 (6)	0.001 (3)	−0.003 (3)	0.051 (3)
C6	0.0490 (13)	0.0655 (14)	0.0756 (15)	0.0063 (11)	0.0003 (11)	0.0450 (13)
C4'	0.056 (3)	0.070 (3)	0.095 (5)	−0.001 (2)	−0.002 (3)	0.040 (4)
C5'	0.064 (3)	0.070 (3)	0.093 (6)	0.001 (3)	−0.003 (3)	0.051 (3)
C7	0.0535 (13)	0.0552 (13)	0.0716 (15)	0.0010 (10)	−0.0045 (11)	0.0398 (12)
C8	0.0552 (14)	0.0628 (14)	0.0701 (15)	0.0056 (11)	0.0030 (11)	0.0386 (12)
C9'	0.067 (6)	0.066 (4)	0.091 (5)	0.010 (4)	−0.020 (5)	0.038 (4)
C10'	0.082 (2)	0.082 (4)	0.077 (6)	−0.002 (2)	−0.014 (3)	0.056 (4)
C11'	0.059 (5)	0.060 (5)	0.105 (6)	0.008 (4)	0.004 (4)	0.058 (5)
C9	0.051 (4)	0.054 (3)	0.067 (3)	−0.001 (3)	−0.005 (3)	0.031 (3)
C10	0.082 (2)	0.082 (4)	0.077 (6)	−0.002 (2)	−0.014 (3)	0.056 (4)
C11	0.051 (3)	0.065 (4)	0.094 (4)	0.005 (3)	−0.003 (3)	0.055 (4)

### Geometric parameters (Å, °)

**Table:**

S1—C1	1.660 (2)	C4'—H4D	0.9700
N1—C1	1.349 (3)	C5'—H5D	0.9700
N1—N2	1.386 (2)	C5'—H5C	0.9700
N1—H1	0.8600	C7—C8	1.488 (3)
N2—C2	1.268 (3)	C7—C11	1.523 (4)
N3—C1	1.349 (3)	C7—C11'	1.528 (4)
N3—N4	1.386 (2)	C8—C9	1.518 (5)
N3—H3	0.8600	C8—C9'	1.532 (6)
N4—C7	1.273 (3)	C8—H8C	0.9700
C2—C6	1.492 (3)	C8—H8B	0.9700
C2—C3	1.502 (3)	C8—H8A	0.9700
C3—C4	1.498 (5)	C8—H8D	0.9700
C3—C4'	1.505 (7)	C9'—C10'	1.507 (7)
C3—H3A	0.9700	C9'—H9C	0.9700
C3—H3B	0.9700	C9'—H9D	0.9700
C4—C5	1.517 (6)	C10'—C11'	1.516 (7)
C4—H4A	0.9700	C10'—H10A	0.9700
C4—H4B	0.9700	C10'—H10B	0.9700
C5—C6	1.536 (5)	C11'—H11C	0.9700
C5—H5A	0.9700	C11'—H11D	0.9700
C5—H5B	0.9700	C9—C10	1.513 (7)
C6—C5'	1.520 (7)	C9—H9A	0.9700
C6—H6C	0.9700	C9—H9B	0.9700
C6—H6B	0.9700	C10—C11	1.523 (7)
C6—H6A	0.9700	C10—H10C	0.9700
C6—H6D	0.9700	C10—H10D	0.9700
C4'—C5'	1.509 (8)	C11—H11A	0.9700
C4'—H4C	0.9700	C11—H11B	0.9700
			
C1—N1—N2	119.09 (16)	C8—C7—C11	109.8 (3)
C1—N1—H1	120.5	N4—C7—C11'	121.1 (4)
N2—N1—H1	120.5	C8—C7—C11'	107.8 (4)
C2—N2—N1	118.56 (17)	C11—C7—C11'	22.1 (4)
C1—N3—N4	121.17 (18)	C7—C8—C9	106.2 (4)
C1—N3—H3	119.4	C7—C8—C9'	103.3 (5)
N4—N3—H3	119.4	C9—C8—C9'	15.73 (7)
C7—N4—N3	114.60 (18)	C7—C8—H8C	111.1
N1—C1—N3	113.33 (19)	C9—C8—H8C	96.0
N1—C1—S1	121.64 (15)	C9'—C8—H8C	111.1
N3—C1—S1	125.03 (16)	C7—C8—H8B	111.1
N2—C2—C6	129.81 (19)	C9—C8—H8B	122.3
N2—C2—C3	120.75 (18)	C9'—C8—H8B	111.1
C6—C2—C3	109.43 (18)	H8C—C8—H8B	109.1
C4—C3—C2	105.4 (4)	C7—C8—H8A	110.4
C4—C3—C4'	18.5 (10)	C9—C8—H8A	110.6
C2—C3—C4'	104.6 (8)	C9'—C8—H8A	125.1
C4—C3—H3A	110.7	H8C—C8—H8A	15.7
C2—C3—H3A	110.7	H8B—C8—H8A	95.6
C4'—C3—H3A	94.4	C7—C8—H8D	110.5
C4—C3—H3B	110.7	C9—C8—H8D	110.5
C2—C3—H3B	110.7	C9'—C8—H8D	97.9
C4'—C3—H3B	126.4	H8C—C8—H8D	120.9
H3A—C3—H3B	108.8	H8B—C8—H8D	14.4
C3—C4—C5	105.0 (6)	H8A—C8—H8D	108.6
C3—C4—H4A	110.7	C10'—C9'—C8	108.7 (10)
C5—C4—H4A	110.7	C10'—C9'—H9C	110.0
C3—C4—H4B	110.7	C8—C9'—H9C	110.0
C5—C4—H4B	110.7	C10'—C9'—H9D	110.0
H4A—C4—H4B	108.8	C8—C9'—H9D	110.0
C4—C5—C6	104.0 (6)	H9C—C9'—H9D	108.3
C4—C5—H5A	111.0	C10'—C9'—H9A	131.9
C6—C5—H5A	111.0	C8—C9'—H9A	117.9
C4—C5—H5B	111.0	H9C—C9'—H9A	65.5
C6—C5—H5B	111.0	H9D—C9'—H9A	43.5
H5A—C5—H5B	109.0	C10'—C9'—H9B	80.7
C2—C6—C5'	104.2 (7)	C8—C9'—H9B	91.0
C2—C6—C5	104.2 (3)	H9C—C9'—H9B	150.6
C5'—C6—C5	24.2 (6)	H9D—C9'—H9B	43.3
C2—C6—H6C	110.9	H9A—C9'—H9B	86.8
C5'—C6—H6C	130.4	C9'—C10'—C11'	99.5 (13)
C5—C6—H6C	110.9	C9'—C10'—H10A	111.9
C2—C6—H6B	110.9	C11'—C10'—H10A	111.9
C5'—C6—H6B	89.0	C9'—C10'—H10B	111.9
C5—C6—H6B	110.9	C11'—C10'—H10B	111.9
H6C—C6—H6B	108.9	H10A—C10'—H10B	109.6
C2—C6—H6A	110.9	C10'—C11'—C7	105.3 (9)
C5'—C6—H6A	110.9	C10'—C11'—H11C	110.7
C5—C6—H6A	89.0	C7—C11'—H11C	110.7
H6C—C6—H6A	23.3	C10'—C11'—H11D	110.7
H6B—C6—H6A	126.6	C7—C11'—H11D	110.7
C2—C6—H6D	110.9	H11C—C11'—H11D	108.8
C5'—C6—H6D	110.9	C10—C9—C8	102.2 (8)
C5—C6—H6D	130.4	C10—C9—H9A	111.1
H6C—C6—H6D	88.2	C8—C9—H9A	111.4
H6B—C6—H6D	23.2	C10—C9—H9B	111.3
H6A—C6—H6D	108.9	C8—C9—H9B	111.4
C3—C4'—C5'	104.6 (11)	H9A—C9—H9B	109.3
C3—C4'—H4C	110.8	C9—C10—C11	106.4 (11)
C5'—C4'—H4C	110.8	C9—C10—H10C	110.5
C3—C4'—H4D	110.8	C11—C10—H10C	110.5
C5'—C4'—H4D	110.8	C9—C10—H10D	110.5
H4C—C4'—H4D	108.9	C11—C10—H10D	110.5
C4'—C5'—C6	104.2 (12)	H10C—C10—H10D	108.7
C4'—C5'—H5D	110.9	C7—C11—C10	98.5 (8)
C6—C5'—H5D	110.9	C7—C11—H11A	112.1
C4'—C5'—H5C	110.9	C10—C11—H11A	112.1
C6—C5'—H5C	110.9	C7—C11—H11B	112.1
H5D—C5'—H5C	108.9	C10—C11—H11B	112.1
N4—C7—C8	129.96 (18)	H11A—C11—H11B	109.7
N4—C7—C11	119.3 (3)		

### Hydrogen-bond geometry (Å, °)

**Table:**

*D*—H···*A*	*D*—H	H···*A*	*D*···*A*	*D*—H···*A*
N3—H3···N2	0.86	2.17	2.558 (3)	108
N1—H1···S1^i^	0.86	2.70	3.547 (3)	170

Symmetry codes: (i) −*x*−1, −*y*+1, −*z*+1.
